# Supplementary material for: Prevalence of Use of Mobile Food Delivery Services During School Hours in the United States: Survey Study
Source: J Med Internet Res. 2026 Sep 4;28:e94675. doi: 10.2196/94675 (PMC13544697; doi:10.2196/94675)
Supplement: Supplementary file 1 [file jmir-v28-e94675-s001.docx]

**Supplementary Table 1. Unweighted percentages of mobile food delivery usage during school and after school. The first percentage in each cell is based on the total sample in this survey and the second percentage is based on the AmeriSpeak Panel sample.**

|  | During school % total sample (n=1,026),  % AmeriSpeak sample (n=722) | | | After school total sample (n=1,024),  AmeriSpeak sample (n=721) | | |
| --- | --- | --- | --- | --- | --- | --- |
| Characteristic | Never | Infrequent | Frequent | Never | Infrequent | Frequent |
| Total | 74%, 80% | 15%, 15% | 10%, 5% | 48%, 56% | 37%, 36% | 15%, 9% |
| Age group |  |  |  |  |  |  |
| 13-14 years old | 78%, 83% | 12%, 12% | 10%, 5% | 54%, 62% | 32%, 30% | 15%, 8% |
| 15-17 years old | 73%, 78% | 17%, 17% | 11%, 5% | 45%, 52% | 40%, 38% | 16%, 9% |
| Sex |  |  |  |  |  |  |
| Male | 70%, 78% | 15%, 15% | 15%, 7% | 45%, 56% | 36%, 33% | 19%, 11% |
| Female | 78%, 81% | 16%, 15% | 6%, 4% | 50%, 55% | 38%, 37% | 12%, 7% |
| Race/Ethnicity |  |  |  |  |  |  |
| White, non-Hispanic | 81%, 88% | 11%, 9% | 9%, 3% | 57%, 66% | 31%, 30% | 12%, 4% |
| Black, non-Hispanic | 49%, 49% | 28%, 30% | 23%, 20% | 17%, 18% | 49%, 52% | 33%, 30% |
| Hispanic | 70%, 76% | 19%, 21% | 10%, 3% | 43%, 52% | 41%, 38% | 16%, 9% |
| Other/2+/Asian-Pacific Islander, non-Hispanic | 82%, 84% | 14%, 12% | 4%, 4% | 50%, 55% | 40%, 37% | 10%, 9% |
| Household Income |  |  |  |  |  |  |
| Less than $30,000 | 67%, 65% | 24%, 26% | 8%, 8% | 44%, 42% | 43%, 44% | 13%, 14% |
| $30,000 to under $60,000 | 73%, 73% | 16%, 17% | 11%, 10% | 44%, 50% | 38%, 36% | 18%, 14% |
| $60,000 to under $100,000 | 78%, 84% | 14%, 12% | 8%, 4% | 49%, 58% | 37%, 33% | 13%, 9% |
| $100,000 or more | 76%, 88% | 12%, 11% | 12%, 2% | 50%, 63% | 34%, 32% | 16%, 4% |
| Regions |  |  |  |  |  |  |
| Northeast | 67%, 71% | 19%, 25% | 14%, 4% | 41%, 52% | 40%, 44% | 19%, 4% |
| Midwest | 83%, 88% | 11%, 8% | 5%, 3% | 56%, 63% | 35%, 31% | 10%, 7% |
| South | 69%, 74% | 18%, 19% | 13%, 8% | 41%, 47% | 39%, 39% | 20%, 14% |
| West | 75%, 80% | 14%, 15% | 11%, 5% | 51%, 59% | 36%, 34% | 13%, 8% |
| Parents’ Highest Education in Household |  |  |  |  |  |  |
| Less than high school | 67%, 68% | 30%, 27% | 4%, 5% | 50%, 52% | 32%, 30% | 18%, 17% |
| High school | 66%, 69% | 21%, 22% | 12%, 10% | 37%, 39% | 42%, 44% | 22%, 18% |
| Some college, vocational/tech school | 76%, 76% | 16%, 17% | 8%, 6% | 47%, 52% | 40%, 40% | 14%, 9% |
| College or higher degrees | 76%, 86% | 12%, 11% | 12%, 3% | 51%, 63% | 34%, 31% | 15%, 6% |
| Geographic characteristics |  |  |  |  |  |  |
| Non-Metropolitan | 77%, 84% | 13%, 10% | 10%, 6% | 56%, 62% | 30%, 30% | 14%, 8% |
| Metropolitan | 74%, 79% | 16%, 16% | 10%, 5% | 46%, 54% | 38%, 37% | 16%, 9% |
| Device used for this survey |  |  |  |  |  |  |
| Desktop computer | 71%, 85% | 12%, 12% | 17%, 2% | 45%, 64% | 32%, 33% | 23%, 3% |
| Smartphone | 75%, 78% | 17%, 16% | 8%, 6% | 49%, 54% | 38%, 36% | 13%, 10% |
| Tablet | 100%, 100% | 0%, 0% | 0%, 0% | 20%, 50% | 80%, 50% | 0%, 0% |

Distributional statistics are expressed as unweighted percentages for total sample, unweighted percentages for AmeriSpeak sample. Infrequent: <1/week; frequent: >1/week.

**Supplemental Table 2: Frequency distribution of whether mobile food delivery apps are allowed in school by region. The frequencies and percentages shown are unweighted n (weighted percent).**

| Characteristic | Not allowed | Allowed | F (ndf, ddf) | p-value |
| --- | --- | --- | --- | --- |
| Total | 722 (73.4%) | 285 (26.6%) |  |  |
| Region |  |  | 1.830 (2.8335, 2907.2063) | *P*=0.143 |
| Northeast | *94 (77.9%)* | 31 (22.1%) |  |  |
| Midwest | *209 (75.6%)* | 77 (24.4%) |  |  |
| South | *263 (75.1%)* | 97 (24.9%) |  |  |
| West | *156 (65.5%)* | 80 (34.5%) |  |  |
| West vs Other regions |  |  | 5.508  (1, 1026) | *P*=0.019 |
| West | *156 (65.5%)* | 80 (34.5%) |  |  |
| Not West | *566 (75.8%)* | 205 (24.2%) |  |  |

ndf: numerator degrees of freedom; ddf: denominator degrees of freedom.

**Supplemental Table 3: Frequency distribution of reasons for not using food delivery apps among students who report never using the service by region.**

| Characteristic | Too expensive | Not allowed by school or other reasons | F (ndf, ddf) | p-value |
| --- | --- | --- | --- | --- |
| Total | 139 (19.7%) | 624 (80.3%) |  |  |
| Region |  |  | 4.884 ( 2.9131, 2219.8127) | *P*= 0.002 |
| Northeast | *11 (15.4%)* | 74 (84.6%) |  |  |
| Midwest | *40 (15.6%)* | 205 (84.4%) |  |  |
| South | *36 (15.5%)* | 217 (84.5%) |  |  |
| West | *52 (32.5%)* | 128 (67.5%) |  |  |
| Region (Binary) |  |  | 14.103  (1, 762) | *P<0.001* |
| West | *52 (32.5%)* | 128 (67.5%) |  |  |
| Not West | *87 (15.5%)* | 496 (84.5%) |  |  |

ndf: numerator degrees of freedom; ddf: denominator degrees of freedom.

**Supplemental Table 4: Frequency distribution of reasons for not using food delivery apps among students who report never using these services for reasons other than school rules by region.**

| Characteristic | Too expensive | Reasons other than school rules or costs | F (ndf, ddf) | p-value |
| --- | --- | --- | --- | --- |
| Total | 139 (41.7%) | 189 (58.3%) |  |  |
| Region |  |  | 2.737 (2.9674, 970.3422) | *P*= 0.043 |
| Northeast | *11 (35.0%)* | 22 (65.0%) |  |  |
| Midwest | *40 (31.4%)* | 68 (38.6%) |  |  |
| South | *36 (38.7%)* | 54 (61.3%) |  |  |
| West | *52 (56.4%)* | 45 (43.6%) |  |  |
| West vs other regions |  |  | 7.765  (1, 327) | *P*=0.006 |
| West | *52 (56.4%)* | 45 (43.6%) |  |  |
| Not West | *87 (35.5%)* | 144 (64.5%) |  |  |

ndf: numerator degrees of freedom; ddf: denominator degrees of freedom.
